# Supplementary material for: Conductive Silver/Carbon Fiber Films for Rapid Detection of Human Coronavirus
Source: Polymers (Basel). 2022 May 12;14(10):1983. doi: 10.3390/polym14101983 (PMC9144029; doi:10.3390/polym14101983)
Supplement: Supplementary file 1 [file polymers-14-01983-s001.zip › polymers-1663439-supplementary.pdf]

Supplementary Materials

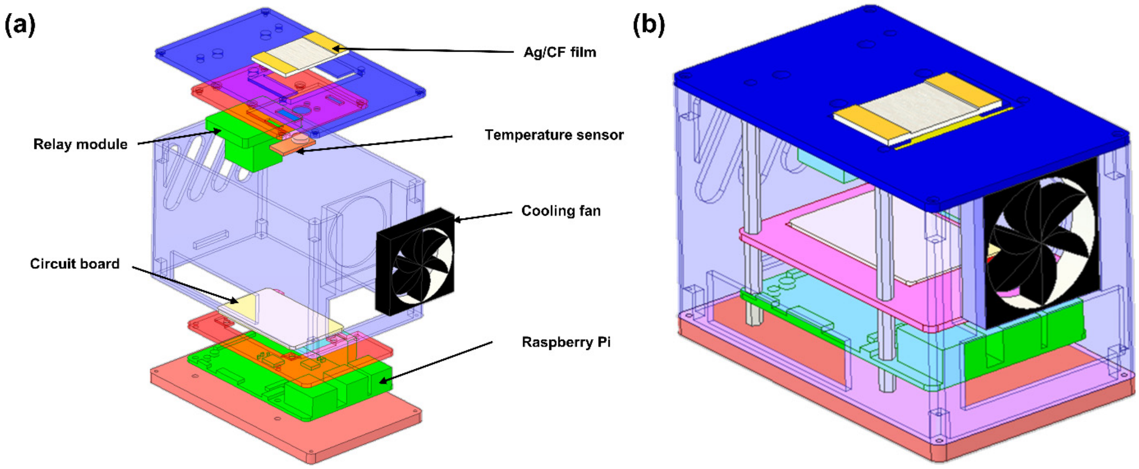

Figure S1. 3D CAD drawings of the Ag/CF film-based thermal cycler. **(a)** Exploded view of the thermal cycler. **(b)** CAD drawing of the assembled thermal cycler.

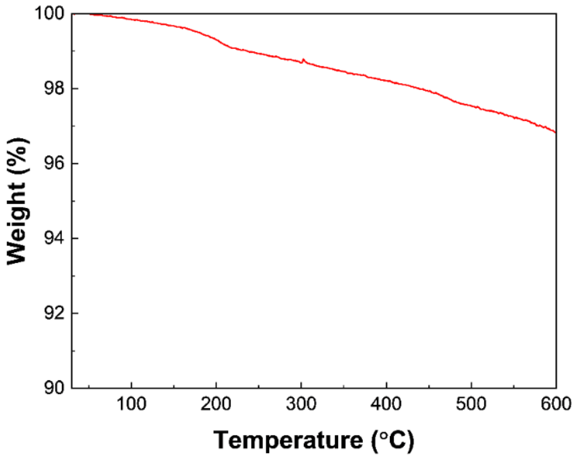

Figure S2. TGA analysis of the Ag/CF film.

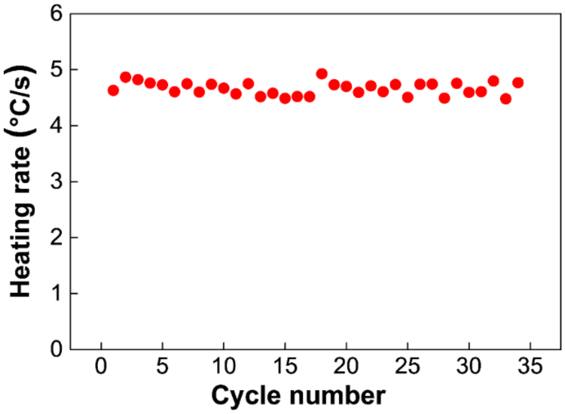

Figure S3. Graph representing average heating rates during 35 cycles of PCR.

Table S1. Sequence of the forward and reverse primers as well as the probe used for multiplex detection of human coronavirus.

Virus	Name	Sequence 5′-3′
OC43	Forward primer	CCCAAGTAGCGATGAGGCTA
	Reverse primer	GTAACCCTGAGGGAGTACCG
	Probe	[FAM] CCGACTAGGTTTCCGCCTGGCA [BHQ1]
229E	Forward primer	CAACAAGCTCCAACAGGCAT
	Reverse primer	GCACGGCAACTGTCATGTAT
	Probe	[TAMRA] AGCACGCCGCTCAGCAAGGT[BHQ2]
NL63	Forward primer	GTTGCTGCTGTTACTTTGGC
	Reverse primer	CTCTCTGGTAGGAACACGCT
	Probe	[Cyanine 5] AGCCTCTTTCTCAACCCAGGGCT [BHQ2]

Table S2. Comparison between the Ag/CF film-based thermal cycler and commercially available thermal cyclers.

	LightCycler	T100 thermal cycler	mini8 thermal cycler	Ag/CF film-based thermal cycler
**Cost (USD)**	15,000	4,912	650	200
**Dimension (mm)**	400×400×530	260×470×230	51×127×102	71×115×76
**Maximum heating rate (°C/sec)**	4.4	5	2.4	4.8
**Power consumption (W)**	600	700	65	5
